# Endoscopic hemostasis followed by preventive transarterial embolization in high-risk patients with bleeding peptic ulcer: 5-year experience

**DOI:** 10.1186/s13017-019-0264-z

**Published:** 2019-09-10

**Authors:** Aleksejs Kaminskis, Patricija Ivanova, Aina Kratovska, Sanita Ponomarjova, Margarita Ptašņuka, Jevgenijs Demičevs, Renate Demičeva, Viesturs Boka, Guntars Pupelis

**Affiliations:** 1 Riga East University Hospital, University of Latvia, Riga, Latvia; 2 Riga Stradins University, Riga, Latvia; 3 University of Latvia, Riga, Latvia

**Keywords:** Non-variceal gastrointestinal bleeding, Transarterial embolization, Preventive

## Abstract

**Background:**

Upper gastrointestinal bleeding (UGIB) due to peptic ulcer disease is one of the leading causes of death in patients with non-variceal bleeding, resulting in up to 10% mortality rate, and the patient group at high risk of rebleeding (Forrest IA, IB, and IIA) often requires additional therapy after endoscopic hemostasis. Preventive transarterial embolization (P-TAE) after endoscopic hemostasis was introduced in our institution in 2014. The aim of the study is an assessment of the intermediate results of P-TAE following primary endoscopic hemostasis in patients with serious comorbid conditions and high risk of rebleeding.

**Methods:**

During the period from 2014 to 2018, a total of 399 patients referred to our institution with a bleeding peptic ulcer, classified as type Forrest IA, IB, or IIA with the Rockall score ≥ 5, after endoscopic hemostasis was prospectively included in two groups—P-TAE group and control group, where endoscopy alone (EA) was performed. The P-TAE patients underwent flow-reducing left gastric artery or gastroduodenal artery embolization according to the ulcer type. The rebleeding rate, complications, frequency of surgical interventions, transfused packed red blood cells (PRBC), amount of fresh frozen plasma (FFP), and mortality rate were analyzed.

**Results:**

From 738 patients with a bleeding peptic ulcer, 399 were at high risk for rebleeding after endoscopic hemostasis. From this cohort, 58 patients underwent P-TAE, and 341 were allocated to the EA. A significantly lower rebleeding rate was observed in the P-TAE group, 3.4% vs. 16.2% in the EA group; *p* = 0.005. The need for surgical intervention reached 10.3% vs. 20.6% in the P-TAE and EA groups accordingly; *p* = 0.065. Patients that underwent P-TAE required less FFP, 1.3 unit vs. 2.6 units in EA; *p* = 0.0001. The mortality rate was similar in groups with a tendency to decrease in the P-TAE group, 5.7% vs. 8.5% in EA; *p* = 0.417.

**Conclusion:**

P-TAE is a feasible and safe procedure, and it may reduce the rebleeding rate and the need for surgical intervention in patients with a bleeding peptic ulcer when the rebleeding risk remains high after primary endoscopic hemostasis.

## Background

The reported incidence of UGIB in the USA and other countries is still between 48 and 160 cases per 100,000 adults per year, reaching a 14% associated mortality, increased hospital admissions and hospitalization costs [1, 2]. All improvements in the medical and endoscopic treatments are not sufficiently effective in treating the aging population with comorbid conditions that often have concomitant treatment with non-steroidal anti-inflammatory or anti-clotting drugs [3]. Peptic ulcer bleeding is seen predominantly among the elderly, with 68% of patients over the age of 60 and 27% over the age of 80 [2]. Elderly people with multiple comorbidities are among those who may fail endoscopic hemostasis and are poor candidates for surgery [4]. Several options are recommended when rebleeding happens, including emergent repeated endoscopy or surgical intervention [5, 6]. TAE has been successfully used for bleeding control, especially in old and multimorbid patients [7]. The preventive mode of transarterial embolization (TAE) has been used successfully as an additional option to decrease the rebleeding rate after endoscopic hemostasis [8]. The goal of P-TAE is a reduction of flow in the ulcer area by embolization of large vessels, such as the left gastric artery or gastroduodenal artery, secondary to ulcer localization in the gastric fundus, antral, pyloric, or duodenal part avoiding a superselective embolization of the vessel feeding the ulcer. This method is technically easier to perform, and it allows avoiding ischemic complications. In a large study analyzing more than 1500 hospital admissions, TAE or surgery was necessary for 5.4% patients; half of them were operated on and another half underwent TAE. A significant part of TAE was done in a preventive mode, reaching a 12.5% mortality rate after TAE and 25.6% after surgery, while the rebleeding rate was 25% after TAE and 16.3% after surgery. The authors conclude that TAE should be the preferred hemostatic method when endoscopy fails [9]; other authors report similar conclusions [10, 11]. Preventive TAE after primary endoscopic hemostasis has been practiced in our institution since 2014. The reduction in the rebleeding rate after preventive TAE encouraged us to continue using this method. The aim of the study is an assessment of the intermediate results of preventive TAE following primary endoscopic hemostasis in patients with serious comorbid conditions and high rebleeding risk.

## Methods

The primary outcome of the study is the rebleeding rate, and the secondary outcomes are the complication rate and mortality. The preparation of the study included an analysis of the medical charts of 922 patients who were emergently admitted to the Riga East University Hospital with UGIB during the period from 2014 to 2018. A bleeding peptic ulcer classified as Forrest Ia, Ib, IIa, or IIb and the Rockall score ≥ 5 after primary endoscopic hemostasis were the main criteria for the inclusion in the study. P-TAE was considered for patients who were at high risk for postoperative mortality due to their age and comorbidities. Those who underwent the preventive mode of TAE were included in the P-TAE group. The control EA group consisted of similar patients who only underwent endoscopic hemostasis or patients who did not agree to undergo P-TAE with a similar prognosis of high rebleeding risk after endoscopic hemostasis and similar comorbid conditions. The exclusion criteria included a hemodynamically unstable patient with a high dependency for ICU support, an increased risk of severe complications associated with the duration of TAE, and a systemic introduction of contrast media. The participants were enrolled and assigned to their treatment by the consensus among the consultant surgeon, consultant radiologist, and duty endoscopy specialist. Endoscopic combination therapy (injection of diluted adrenaline 1:10,000, treatment with a heater probe, and/or hemoclip) followed by a 72-h infusion of esomeprozole (80 mg bolus followed by 8 mg/h) was applied to all patients [5]. Patients were closely monitored at ICU.

### Technical approach

All patients with evidence of UGIB after admission underwent endoscopic combination therapy followed by a 72-h infusion of esomeprazole. Those who had high rebleeding risk after primary endoscopic hemostasis were considered for additional hemostasis including the surgical or repeated endoscopic approach. According to the consensus of the consultant surgeon, consultant radiologist, and duty endoscopy specialist, the alternative TAE approach was recommended to a selective group of patients. Patients who were at high risk for rebleeding and were not candidates for emergent surgical intervention due to a critical comorbid status were selected for P-TAE within 24 h of a successful primary endoscopic hemostasis. Visceral angiography and TAE were performed by the invasive radiologist. The technical goal of P-TAE was the embolization of the left gastric artery or gastroduodenal artery (depending on the ulcer localization) within 24 h of endoscopic hemostasis, achieving a decrease of the arterial flow in the tissue beneath the ulcer. In cases with the ulcer localized in the smaller or greater curvature or the gastric fundus, the left gastric artery was obliterated; in cases of gastric antral, pyloric, or duodenal ulcers, the gastroduodenal artery was embolized [8, 12]. Rebleeding was defined as the presence of hematemesis, blood from the nasogastric tube, or melena associated with a fall in hemoglobin of more than 0.8 g/dl (not explained by hemodilution) or arterial hypotension after primary endoscopy. If therapeutic endoscopy was insufficient to control the bleeding (technically difficult primary therapeutic endoscopy or signs of exsanguination), TAE or surgical hemostasis could be performed without being preceded by repeat endoscopy. The complication rate, recurrence of bleeding, and the need for repeat endoscopic therapy or surgery were the variables for the statistical analysis in groups. Hospital stay, including the duration of the intensive care stay, and in-hospital mortality rate among the groups were analyzed. The study was approved by the local research ethics committee and followed the Declaration of Helsinki. All authors had access to the study data and have reviewed and approved the final manuscript.

### Statistical analysis

Interval data are presented as the mean value with the standard deviation (mean ± SD). A comparison of linear data was performed using the Mann-Whitney *U* test. A comparison of nominal data was performed using Pearson’s chi-squared test. *P* < 0.005 was considered as statistically significant with a confidence interval of 95%. The statistical analysis of data was performed with the IMB SPSS Statistics version 23.

## Results

### Cohort

During the 5-year period, 922 patients were presented to Riga East University Hospital with UGIB. Of all, 738 patients had a bleeding peptic ulcer, and 399 of them had an ulcer classified as Forrest Ia, Ib, IIa, or IIb, and the Rockall score ≥ 5, corresponding to the high rebleeding risk category. The mean age of patients in the P-TAE group was 70.9 ± 15.5 years vs. 66.4 ± 14.5 years in the EA group (*p* = 0.028) (Table 1). There was no statistically significant difference in the gender and comorbid conditions of the patients, including cardiac, pulmonary, renal, or cerebral disease and the presence of cancer. The mean ASA score in the P-TAE group was 4.4 ± 0.6, and in the EA group, the ASA score was 3.7 ± 0.7 (*p* ≤ 0.001).

**Table 1:** Patient characteristics

Characteristics	P-TAE	EA	*p* value
	*n* = 58	*n* = 341	
Age, years, mean ± SD	70.9 ± 12.5	66.4 ± 14.5	0.028
Gender/male, no of pts	32 (55.7%)	190 (55.2%)	0.938
Comorbidities, no of pts	41 (70.7%)	262 (76.8%)	0.321
Heart disease, no of pts	39 (67.2%)	240 (70.4%)	0.644
Kidney disease, no of pts	9 (15.5%)	58 (17.0%)	0.852
Liver disease, no of pts	5 (8.6%)	29 (8.5%)	0.982
Cancer, no of pts	4 (6.9%)	18 (5.3%)	0.622
Diabetes mellitus, no of pts	9 (15.8%)	61 (17.9%)	0.680
Respiratory disease, no of pts	6 (10.3%)	48 (14.1%)	0.434
Cerebral disease, no of pts	16 (27.6%)	70 (20.5%)	0.231
ASA score, points, mean ± SD	4.4 ± 0.6	3.7 ± 0.7	< 0.001

### Preventive embolization

In total, 52 (13%) patients experienced rebleeding after primary treatment. Definitive hemostasis was achieved surgically in 39 patients, with TAE in two and repeated endoscopy in 11 patients.

From the whole cohort, 58 (14.5%) patients had critical comorbidities, severely increasing the risk of surgery in case of rebleeding, and they were selected for preventive TAE after a successful primary endoscopic hemostasis (P-TAE group). The control group (EA) consisted of 341 patients statistically selected by the SPSS 21 program complying with the same criteria and comorbid condition status as the P-TAE group.

### Endoscopic findings

The endoscopic findings revealed a similar type of ulcer size and distribution, with a mean size 304.7 ± 586.9 mm^2^ vs. 126.5 ± 254.6 mm^2^, *p* = 0.073, most commonly Forrest IIb type—37.9% vs. 48.7% (*p* = 0.167). There was no statistically significant difference in hemoglobin levels and erythrocyte counts in the P-TAE and EA groups (Table 2). The median Rockall score was 7 in both groups (*p* = 0.597). No statistically significant difference in any parameters was observed comparing patients who underwent preventive TAE and those who underwent surgery.

**Table 2:** Ulcer characteristics

Characteristics	P-TAE	EA	*p* value
Ulcer size, mm, mean ± SD	304.7 ± 586.9	126.5 ± 254.6	0.073
Forrest IA, no of pts	6 (10.3%)	40 (11.7%)	0.167
Forrest IB, no of pts	8 (13.8%)	53 (15.5%)	0.167
Forrest IIA, no of pts	22 (37.9%)	82 (24.0%)	0.167
Forrest IIB, no of pts	22 (37.9%)	166 (48.7%)	0.167
HGB, g/dL, mean ± SD	8.6 ± 2.6	9.2 ± 3.1	0.176
RBC, ×1012/L, mean ± SD	2.9 ± 0.8	3.1 ± 1	0.197
INR, ratio, mean ± SD	1.3 ± 0.8	1.6 ± 5.7	0.732
Rockall score, points, mean ± SD	6.9 ± 1.4	6.9 ± 1.3	0.597

### Outcomes

The rebleeding rate was lower in the P-TAE group, 3.4% vs. 16.2% (*p* = 0.005) (Table 3). Six patients (10.3%, *p* = 0.065) from the P-TAE group required surgical intervention; in two of them, the indication was recurrent bleeding, and in four, surgical intervention was indicated because of a large-size or high-risk ulcer. Preventive TAE stabilized the patient condition before surgical intervention. From the EA group, 71 patients (20.6%, *p* = 0.065) required surgical intervention, 35 because of recurrent bleeding, 16 because of a large ulcer, and 6 due to failed endoscopic hemostasis (Table 3). Transfusion support was needed for the majority of patients. The mean amount of transfused packed red blood cells (PRBC) was bigger in the P-TAE group than the EA group, *p* = 0.002. The mean amount of transfused FFP was lower in the P-TAE group, *p* = 0.001 (Table 3). No ischemic complications were observed in patients after preventive TAE. A similar mean ICU stay was necessary in both groups (3.5 ± 2.2 vs 4 ± 3.5 (*p* = 0.300)). No difference in the mean hospital stay was observed in both groups (*p* = 0.759). No statistically significant difference was observed in the mortality rates in both groups 5.7% vs. 8.5%, *p* = 0.417 (Table 3). Preventive TAE allowed achieving a significantly lower rebleeding risk than in the control group.

**Table 3:** Outcomes

Characteristics	P-TAE	EA	*p* value
ICU stay days, mean ± SD	3.5 ± 2.2	4 ± 3.5	0.300
Hospital stay days, mean ± SD	10.2 ± 8	9.8 ± 6.8	0.670
Re-bleeding, *n*%	2 (3.4%)	50 (16.2%)	0.005
Mortality, *n*%	3 (5.7%)	28 (8.5%)	0.417
PRBC, units, mean ± SD	6.6 ± 2.2	3.6 ± 2.5	0.002
FFP, units, mean ± SD	1.3 ± 1	2.6 ± 1.7	0.001
Surgery, *n*%	6 (10.3%)	71 (20.6%)	0.065

## Discussion

TAE has become popular in the treatment of non-variceal UGIB in the past two decades. It can be used as a bleeding control method in case of failed endoscopy or as a method to prevent recurrent bleeding after a successful primary endoscopic therapy [9, 13]. In this study, TAE was used as a preventive tool in patients at high risk of recurrent bleeding after primary endoscopy.

The mean age of patients who underwent preventive TAE was 70.9 ± 12.5 years, which is higher than reported by other authors. [9, 14]. Spiliopoulos et al. reported a mean age of 74 years for patients undergoing TAE in their retrospective study [13]. The patient characteristics in the recently reported study by Lau et al. are similar to the results of the present study considering the age and characteristics of the ulcer. In Asian trials, a predominance of male patients is reported; contrary to that, European studies report a proportion of gender similar to our results [9, 13, 14].

Risk assessment is an indisputable part of the management strategy in patients with acute gastrointestinal bleeding. Even considering the latest progress in endoscopic, surgical, and interventional radiology, there is still a rather high rate of rebleeding—up to 20%—as well as deaths ranging from 5 to 10% particularly in unselected patients. Risk factor identification predicting a high risk of rebleeding is one of the ways for outcome improvement in patients who are poor candidates for surgery [10, 11, 15]. Several criteria have been proposed for the stratification of high- and low-risk patients. The criteria include the physiologic response to bleeding: acute upper gastrointestinal bleeding and a substantial loss of intravascular volume, resting tachycardia, hypotension (systolic blood pressure, < 100 mm Hg), or postural changes (an increase in the pulse rate) and the importance of endoscopic findings (ulcer size and completeness of endoscopic hemostasis) [2, 15]. The Glasgow-Blatchford score is widely recommended for the prediction of outcomes and the timing of medical intervention including emergent endoscopy in patients with upper gastrointestinal bleeding [6, 16–20]. The Rockall score is calculated based on the clinical variables indicating the urgency of endoscopic intervention and evidence of stigmata of bleeding [2, 21]. The Glasgow-Blatchford score and the Rockall score are superior considering their sensitivity in predicting the rebleeding rate [15, 17]. Factors like hemoglobin levels, ulcer size, arterial pressure, heart rate, and ASA score were taken into account when making a decision about further therapy after a successful primary endoscopy.

TAE may be used as both a bleeding control method in case of failed endoscopic treatment and a prophylactic method after a successful primary endoscopy [9, 14]. A high risk of recurrent bleeding after primary endoscopic hemostasis and a critical physiologic status associated with serious comorbidities were the characteristic features of the enrolled patient cohort determining the high surgical intervention risk and favoring the less aggressive preventive TAE approach. The evaluation of the study results demonstrated that preventive embolization reduces the recurrent bleeding rate and the need for surgery in our cohort. Recent European and Asian studies also show that patients who underwent prophylactic angiographic embolization had a lower recurrent bleeding rate and need for surgery [14, 22].

The type, size, and localization of the ulcer are very important selection criteria [13]. The incidence of high-risk Forrest Ia, Ib, IIa, and IIb type ulcers is different in several reports. Lau et al. included only patients with Forrest Ia, Ib, and IIa ulcers having more patients with Forrest Ib, but a similar number of patients with Forrest Ia ulcers. Laursen et al. also reported more than a third of patients with Forrest Ib ulcers, Nykanen et al. reported a prevalence of Forrest Ia and Ib ulcers in the majority of patients [9, 14, 23]. In our study, Forrest Ia and Ib ulcers were observed in 27% and Forrest IIa and IIb ulcers in 73% of the included patients. The difference in ulcer presentation could be associated with the number of patients who were referred late and a different interpretation of the endoscopic findings by our endoscopy specialists.

One of the main criteria for high-risk patient selection is the Rockall score. According to our previous study, the Rockall score ≥ 5 was associated with a high risk of recurrent bleeding in patients with Forrest Ia–IIb ulcers [24]. Mille et al. in their study performed preventive embolization in high-risk patients with Forrest Ia–IIc ulcers and the Rockall score ≥ 6 [22]. A similar approach was used for the selection of candidates for preventive TAE in the present study.

Our results showed that patients who underwent preventive TAE required more PRBC than EA (6.6 ± 2.2 units vs. 3.6 ± 1.7 units, *p* = 0.002). It may be explained by a lower hemoglobin level at presentation comparing with the EA. Mille et al. observed similar results in their trial comparing patients who underwent prophylactic TAE (3.9 units) and endoscopic treatment group (1.7 units). They described the endoscopic treatment group as relatively healthier [22]. Lau et al. reported a median of two transfused blood units in both groups. Laursen et al. reported a median of 4.3 blood transfusion units for the TAE group and 4.9 units for the control group, but no statistical difference was observed [14]. However, the transfusion rate of FFP was significantly higher in the EA than in the P-TAE group. The reason is not clear, and the explanation reported by other authors that the control group may have higher INR at presentation time is not consistent with our results showing no difference in the level of INR in groups.

The incidence of recurrent bleeding after preventive embolization is crucial for the outcome. Lau et al. reported a rebleeding rate of 6.2% for patients who underwent preventive embolization and 11.4% after standard treatment without a statistical significance. Laursen et al. reported rebleeding rates of 4% in the group that underwent supplementary TAE and 14% for the control group [14, 23]. Our results demonstrated a significant difference in favor of TAE (3.4% vs. 16.2%, *p* = 0.005). Surgical intervention was the only option remaining for 2 patients who had rebleeding after preventive TAE, significantly less compared to the control group. The ICU stay, hospital stay, and mortality were not different in our study; similar results were reported by Lau et al. and Nykanen et al. [9, 14]. Laursen et al. reported shorter median hospital stays for patients after TAE [23]. Our strategy resulted in a comparable 5.7% and 8.5% mortality rate between groups similar to reports from other authors [9, 14, 23].

### Limitations of the study

The most significant limitation of our study is the lack of randomization because the interventional radiologist and interventional radiology personnel are not available during the night and on weekends. The unequal distribution of patients between both groups is the next major limitation; however, improving availability of invasive radiology service, a randomized trial is justified.

## Conclusion

Preventive TAE is feasible and safe in patients with a bleeding peptic ulcer when the rebleeding risk remains high after endoscopic hemostasis, reducing the rebleeding rate and the need for surgical intervention. Even if surgery is needed due to a high-risk peptic ulcer, preventive TAE allows preparing the patient for the operation by minimizing rebleeding in the pre-operative period. Preventive TAE should be the preferred method in elderly and multimorbid patients who are poor candidates for surgery and have a high risk of post-operative mortality.

## Data Availability

Not applicable.

## Abbreviations

EA: Endoscopy alone group
FFP: Fresh frozen plasma
HGB: Hemoglobin
ICU: Intensive care unit
INR: International normalized ratio
PRBC: Packed red blood cells
P-TAE: Preventive transarterial embolization group
RBC: Red blood cells
TAE: Transarterial embolization
UGIB: Upper gastrointestinal bleeding
